# Strong Valence Electrons Dependent and Logical Relations of Elemental Impurities in 2D Binary Semiconductor: a Case of GeP_3_ Monolayer from Ab Initio Studies

**DOI:** 10.1186/s11671-019-3135-3

**Published:** 2019-09-09

**Authors:** Suihao Zhang, Rui Li, Xiaonan Fu, Yu Zhao, Chunyao Niu, Chong Li, Zaiping Zeng, Songyou Wang, Congxin Xia, Yu Jia

**Affiliations:** 1 International Laboratory for Quantum Functional Materials of Henan, and School of Physics and Engineering, Zhengzhou University, Zhengzhou, 450001 China; 2 Key Laboratory for Special Functional Materials of Ministry of Education, and School of Materials Science and Engineering, Henan University, Kaifeng, 475001 China; 3 College of Science, Henan University of Technology, Zhengzhou, 450001 China; 4 Shanghai Ultra-Precision Optical Manufacturing Engineering Center and Department of Optical Science and Engineering, Fudan University, Shanghai, 20043 China; 5 College of Physics and Materials, Henan Normal University, Xinxiang, 453007 China

**Keywords:** First-principle calculations, GeP_3_ monolayer, Co-doping, Formation energy

## Abstract

Using first-principle calculations within density functional theory, we investigate the electronic property and stability of substitutionally doped 2D GeP_3_ monolayer with dopants from group III to VI. The conducting properties are found to be dramatically modified by both the doping sites and the number of valence electrons of dopants. Specifically, substitution on Ge site exhibits metal-semiconductor oscillations as a function of the number of valence electrons of dopants, while such oscillations are totally reversed when substitution on P site. Moreover, we also study the case of co-doping in GeP_3_, showing that co-doping can produce a logical “AND” phenomenon, that is, the conducting properties of co-doped GeP_3_ can be deduced via a simple logical relation according to the results of single doping. Finally, we investigate the formation energy of dopants and find that the electron-hole and hole-hole co-doped systems are much more energetically favorable due to the Coulomb attraction. Our findings not only present a comprehensive understanding of 2D doping phenomenon, but also propose an intriguing route to tune the electronic properties of 2D binary semiconductors.

## Introduction

Since the discovery of graphene [1, 2], the family of two-dimensional (2D) crystals such as transition metal dichalcogenides (TMDs) [3], silicene [4], germanene [5], phosphorene [6], tellurene [7], and so on have attracted great attention due to their unique electrical, optical, and magnetic properties [8–10]. For example, graphene behaves like massless Dirac fermions, which gives rise to ultimate high-charge carrier mobility [11, 12]. Thus, it is promising to support the 2D quantum spin Hall effect, enhanced thermoelectricity, superconductivity [13], and even the quantum anomalous Hall effect [14–16]. Combined with the growing number of available crystal structure databases, modern computational tools have been used to explore newly undiscovered 2D materials. Up to now, more than 1000 2D materials are predicted and some of them are fabricated in experiment [17–19], becoming an interesting field in physical, chemical, and material science. Such fundamental studies and explorations of 2D materials also boost their great potential applications to the sensing field [20–25].

Recently, Jing et al. reported new 2D material-GeP_3_ monolayer, which has higher chemical stability than BP monolayer and possesses excellent electronic and optical properties. Moreover, the 2D GeP_3_ monolayer appears to have a semiconducting property due to the strong interlayer quantum confinement. They found that the GeP_3_ monolayer exhibits a moderate and tunable band gap about 0.55 eV [26]. Based on the high capacity and good cyclic stability, GeP_3_ thin film is proposed for lithium-ion batteries as a promising anode [27]. Li et al. also investigated the GeP_3_ nanoribbon and discovered the band gaps can exhibit even-odd oscillations with the width increase [28].

Doping is a practical strategy to fundamentally tune the electronic and magnetic properties of the host 2D-layered materials [29]. Besides, it breaks the limitation of a single material in the applications of many fields and devices. As we know, the 2D monolayer semiconductor can result in remarkably enhanced electron-electron interactions which have been demonstrated to generate large band gap renormalization and exciton from both many-body theoretical calculations and experiments [30, 31]. Compared with doping in bulk semiconductors, doping in 2D ones is also expected to exhibit some abnormal behaviors due to the strong electron confinement effect, i.e., graphene doped with boron or nitrogen is possible to open a small band gap at the Dirac point, and the band gap of graphene also can be opened effectively around K (or K’) points by introducing small BN domains [32]. Band gaps of black phosphorene show an oscillating behavior by doping different elements with even or odd numbers of valence electron [33, 34]. In this work, we try to extend the investigation of doping elements of group IV–V in 2D binary GeP_3_ monolayer semiconductor.

Here, we performed the systematic studies of the substitutionally doped GeP_3_ monolayer with the dopants from group III to VI. The electronic properties of doped systems will be dramatically affected by both the number of valence electrons of dopants and the doping sites. The central grains are (1) for single dopant, the results are sensitively dependent on the substitution sites and the substitution on two kinds of doping sites will produce totally inverse results. (2) The conducting properties of co-doping can be deduced by a logical operator through the ones of the single dopant. Additionally, the calculated formation energy of different types of doping suggests that some of them are highly energetically favorable against thermal fluctuation.

## Computational Methods

All our density functional theory calculations within the general gradient approximation are performed using Vienna ab initio Simulation Package [35]. The exchange and correlation terms were described with the Perdew-Burke-Ernzerhof (PBE) functional, and the projector augmented wave potential was employed to describe the electron-ion interaction [36–38]. The doped GeP_3_ monolayer was modeled in a periodic 2 × 2 supercell containing 32 atoms, and a larger supercell of 3 × 3 was also used to check our results. A vacuum space of about 20 Å along the *z* direction was adopted in order to eliminate the interaction between neighboring layers. For the single doping, one Ge or P atom was substituted by a dopant from group III (IV, V, and VI). The geometrical structures are determined by comparing with the reported results, including lattice constant and electronic property of host GeP_3_ monolayer. In the doping systems, all the atoms in the supercells are allowed to relax until the Hellmann-Feynman force is less than 0.02 eVÅ^−1^, but the lattice constants of the surface cells are fixed during the atom relaxation. A kinetic energy cutoff of about 600 eV and 6 × 6 × 1 *k*-meshes were used, respectively [39].

In order to check the availability of the dopants in the GeP_3_ monolayer, the formation energy (*E*_f_) of dopants *X* (*X* = group III–VI) is calculated according to the two following formulas. For single dopant, we have the following:

1 $$ {\mathrm{E}}_{\mathrm{f}}\left(\mathrm{Ge}{\mathrm{P}}_3:\mathrm{X}\right)=\mathrm{E}\left(\mathrm{Ge}{\mathrm{P}}_3:\mathrm{X}\right)-\mathrm{E}\left(\mathrm{Ge}{\mathrm{P}}_3\right)-{E}_{\mathrm{X}}+{E}_{\mathrm{i}} $$

and for the co-doping system, a similar formula is used:

2 $$ {\mathrm{E}}_{\mathrm{f}}\left(\mathrm{Ge}{\mathrm{P}}_3:\mathrm{XY}\right)=\mathrm{E}\left(\mathrm{Ge}{\mathrm{P}}_3:\mathrm{XY}\right)-\mathrm{E}\left(\mathrm{Ge}{\mathrm{P}}_3\right)-{E}_{\mathrm{X}}-{E}_{\mathrm{Y}}+{E}_{\mathrm{i}}+{E}_{\mathrm{j}} $$

where *E*_f_(GeP_3_ : *X*) and *E*(GeP_3_) are the total energies of the X-doped and intrinsic GeP_3_ monolayer with the same supercell. *E*(GeP_3_ : *XY*) is the total energies of the XY co-doped system, *E*_X_ and *E*_Y_ are the atomic energies of dopants X or Y refereed to their corresponding bulk structures, and *E*_i_, *E*_j_ are the energies of substituted atoms where i and j indicate the Ge or P atom, respectively [40, 41].

## Results and Discussions

### Even-Odd Oscillations for Single Element Doping Systems

**Fig. 1:**
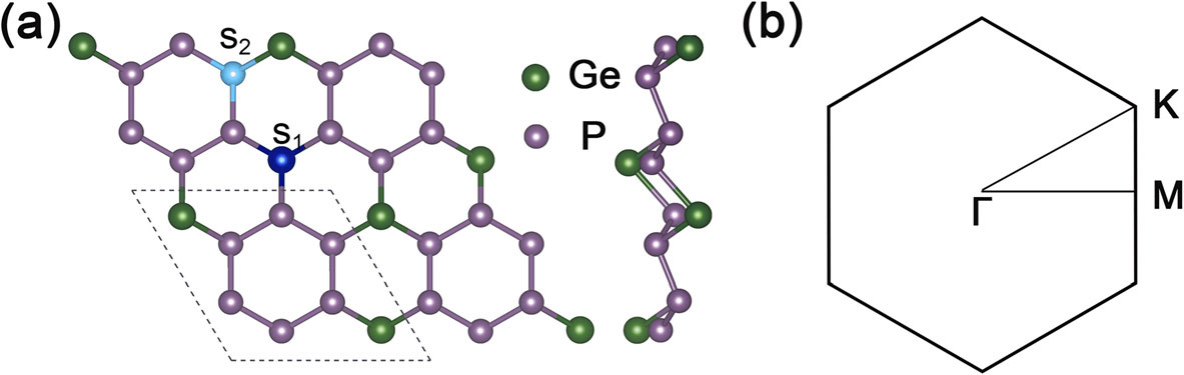
Geometrical structure and Brillouin zone of GeP_3_. **a** Top and side views of the optimized geometry of GeP_3_ with a 2 × 2 supercell. The dotted line presents the unit cell of GeP_3_ monolayer, S_1_ represents the site of substituting position of Ge site, and S_2_ represents the site of substituting position P atom. **b** the 2D Brillouin zone of GeP_3_ monolayer

Figure 1a shows the top and side view of the structure of the GeP_3_ 2 × 2 supercell, and Fig. 1b is the corresponding 2D Brillouin zone of GeP_3_ monolayer. The optimized lattice constants of GeP_3_ monolayer are $\mathrm{a}=\mathrm{b}=6.96\ {\AA}$, and the calculated band gap is about 0.26 eV, which are in good agreement with other theoretical calculations.

**Fig. 2:**
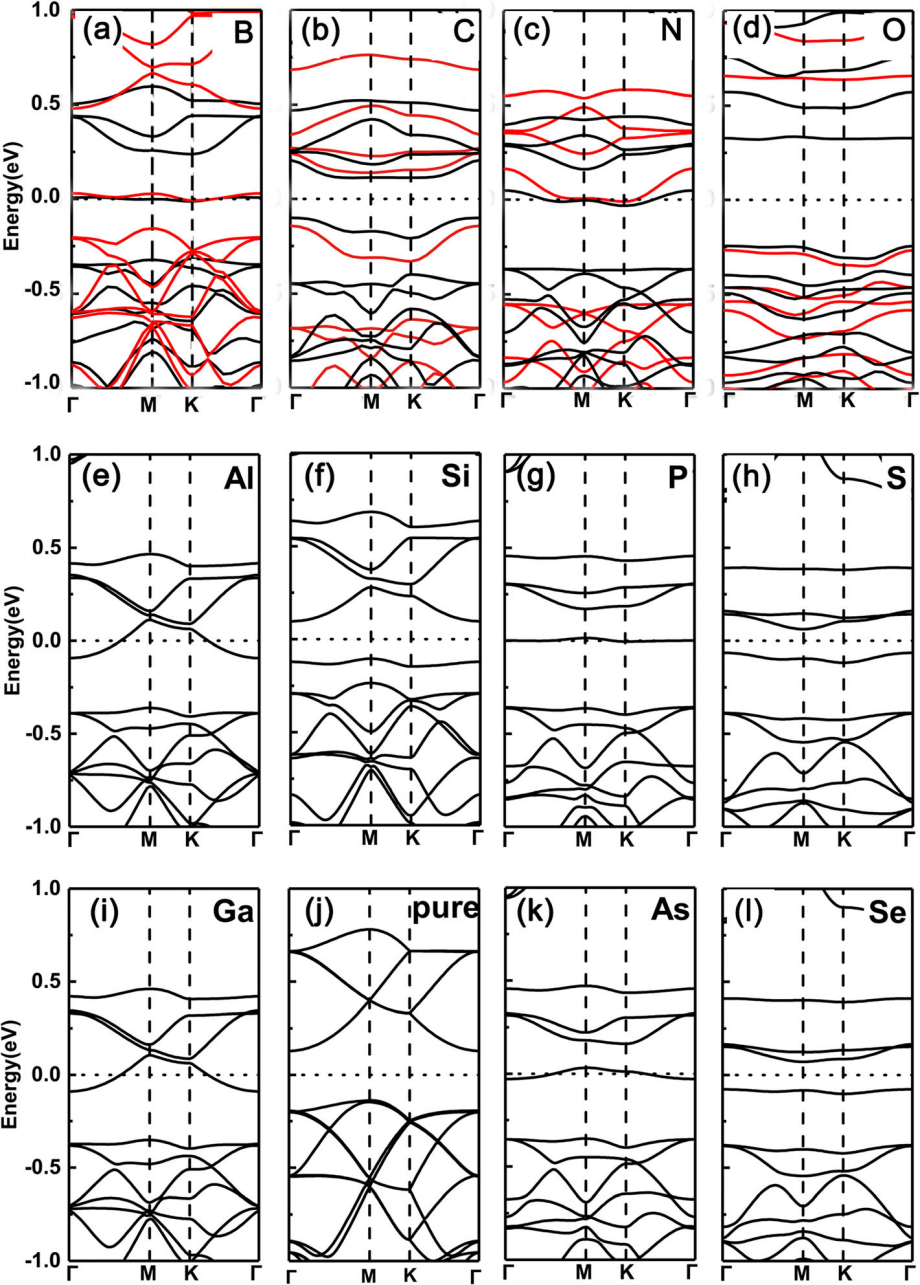
Band structures of the various dopants in GeP_3_ monolayer with substituting Ge atom. **a** B, **b** C, **c** N, **d** O, **e** Al, **f** Si, **g** P, **h** S, **i** Ga, **j** Pure GeP_3_, **k** As, **l** Se. Calculated band structures for a $\mathsf{2}\times \mathsf{2}$ supercell with various dopants in GeP_3_ monolayer from group III to VI, with substituting Ge atom, respectively, together with that of pure GeP_3_ monolayer. Both the PBE and HSE06 functionals are employed in the topmost row

Firstly, we plotted the band structures of single element-doped GeP_3_ monolayer with a substituting Ge atom (here, we choose B, C, N, O, Al, Si, P, S, Ga, As, and Se as dopants). The results are shown in Fig. 2a–l, respectively. We can see clearly the Fermi level shifts up and crosses the conduction bands for group V (N, P, As) due to one more electron dopant, while for group III (B, Al, Ga) dopants due to one less electron, the one shifts down and crosses the valence bands. For example, in Fig. 2f and j, their valence band maximum just corresponds to the partially occupied bands shown in Fig. 2e and i. However, for group IV (C, Si, and Ge) and VI (O, S, and Se) dopants, because of the same or two more electrons as Ge atom, the systems exhibit a semiconductor feature. Such tuning of semiconducting to metallic transition stems from the occupation of the number of valence electrons, namely odd (even) valence electrons occupation leads to metallic (semiconducting) properties.

To confirm the validity of the above results derived from PBE functionals, we also employ the hybrid density functional (HSE06) functionals to check the doped systems of the topmost row. It is clear that PBE functionals indeed give the mistakes of band gaps due to the underestimation. However, in our studied systems, all of them have sizeable gaps, this means that the mistakes between metallic or semiconducting properties caused by the PBE functionals usually will not happen (This is because in some small band gaps of semiconductors, PBE functionals usually give rise to the mistake between conducting and metallic properties). Moreover, in our study, what we are concerned with is the metallic or semiconducting characteristics, instead of the specific values of band gaps. Compared with the gaps derived from PBE functionals, the gaps from HSE06 functionals enlarge clearly. Even with these, the metal-semiconductor oscillations remain intact. Therefore, the central ingredients drawn based on PBE functionals are reliable.

**Fig. 3:**
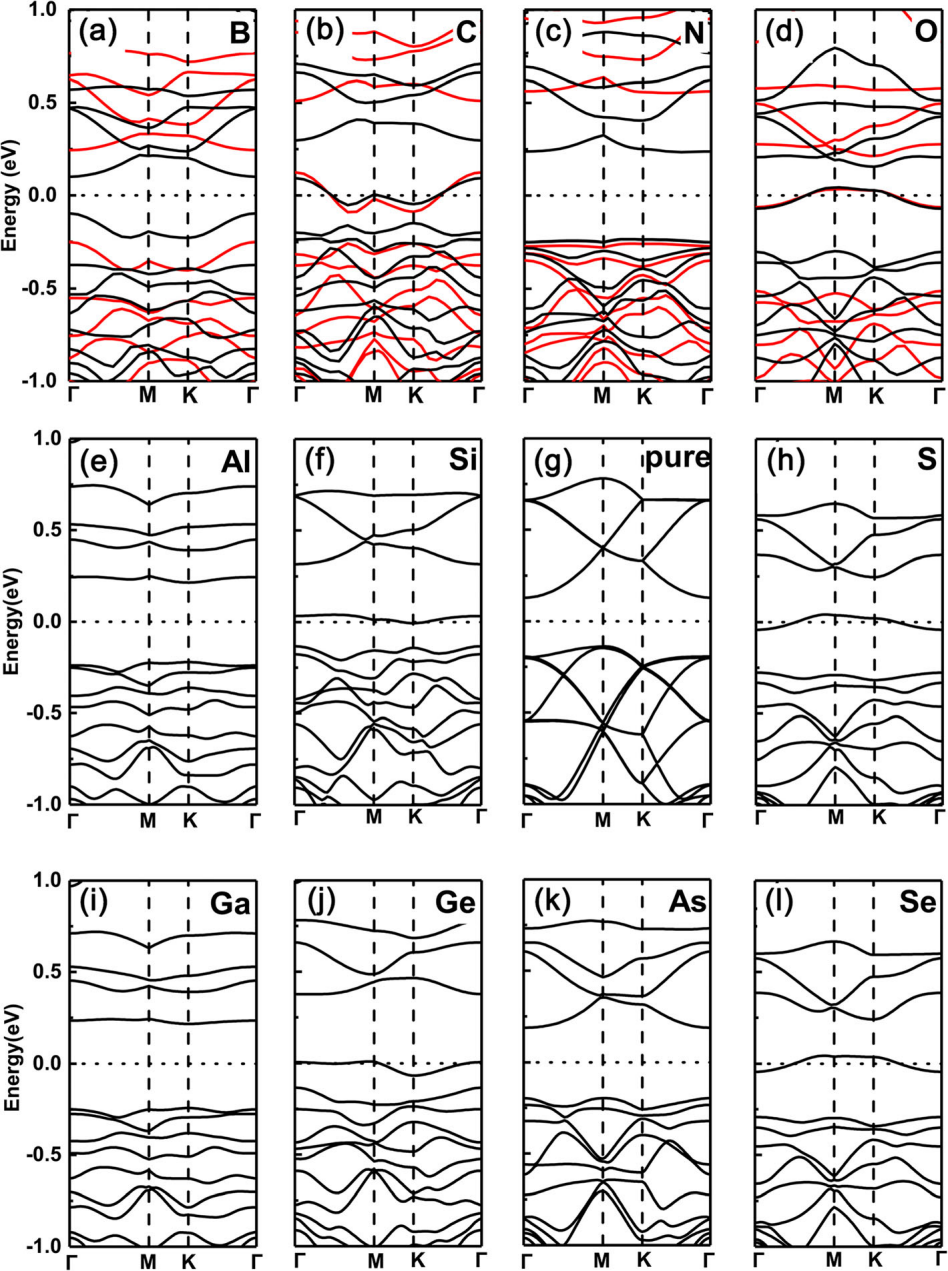
Band structures of the various dopants in GeP_3_ monolayer with substituting P atom. **a** B, **b** C, **c** N, **d** O, **e** Al, **f** Si, **g** P, **h** S, **i** Ga, **j** Pure GeP_3_, **k** As, **l** Se. Calculated band structures for a $\mathsf{2}\times \mathsf{2}$ supercell with various dopants in GeP_3_ monolayer from group III to VI, with substituting P atoms, respectively, together with that of pure GeP_3_ monolayer. Both the PBE and HSE06 functionals are employed in the topmost row

However, in sharp contrast, the cases of substituting P atoms by the same dopants are totally reversed, as shown in Fig. 3a–l, respectively. That is, for group V (N, As) and group III (B, Al, Ga) dopants, the doped systems remain semiconducting property, while for group IV (C, Si, Ge) and VI (O, S, Se) dopants, the ones change to metallic feature (here, the same trend is also found between PBE and HSE06 functionals). This is because the valence electrons keep the same (two less) as (than) the intrinsic GeP_3_ for group V (group III) dopants, but one less (more) electron for group IV (VI) dopants.

**Fig. 4:**
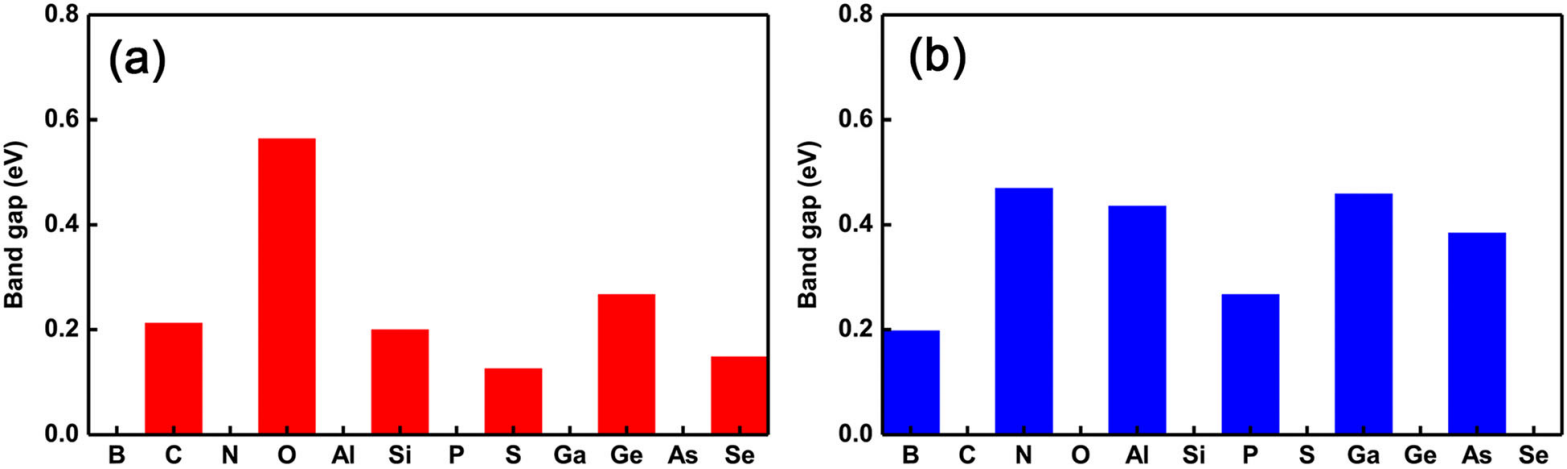
Band gaps of all single-doped systems. Band gaps of doped GeP_3_ monolayers with the different dopants ranging from group V to VI. **a** the substitution of Ge atoms and **b** the substitution P atoms, respectively

To better present the oscillations of semiconducting to metallic properties transition, we plotted the changing trend of the band gap as the different dopants, as shown in Fig. 4a and b, respectively. Clearly, we can see that the transition from semiconducting to metallic properties is drastically reversing. Specifically, metallic (semiconducting)–semiconducting (metallic) oscillations occur in substituting Ge (P) site as the dopants ranging from group III to VI. Besides, we also found an interesting phenomenon which shows that the magnitude of the bandgap nearly keeps the same as intrinsic GeP_3_ monolayer when the dopants have the same valence electrons as the Ge atom. However, when the dopants have two more electrons than the Ge atom, the magnitude of the band gaps change relatively larger. Nevertheless, for the dopants at P sites, regardless of the number of valence electrons, the magnitude of the band gaps always change large relatively. This can be understood by the joint effect of radius of the atom and the available valence electrons, namely dopants with nearly the same (smaller or larger) radius and valence electrons as (than) Ge atom cause relatively smaller (larger) effect on the electronic properties, such as the band gap. This means that one can tune not only the oscillations of semiconducting-metallic transitions, but also can tune the magnitude of the band gap by choosing proper dopants and the different doping sites.

**Fig. 5:**
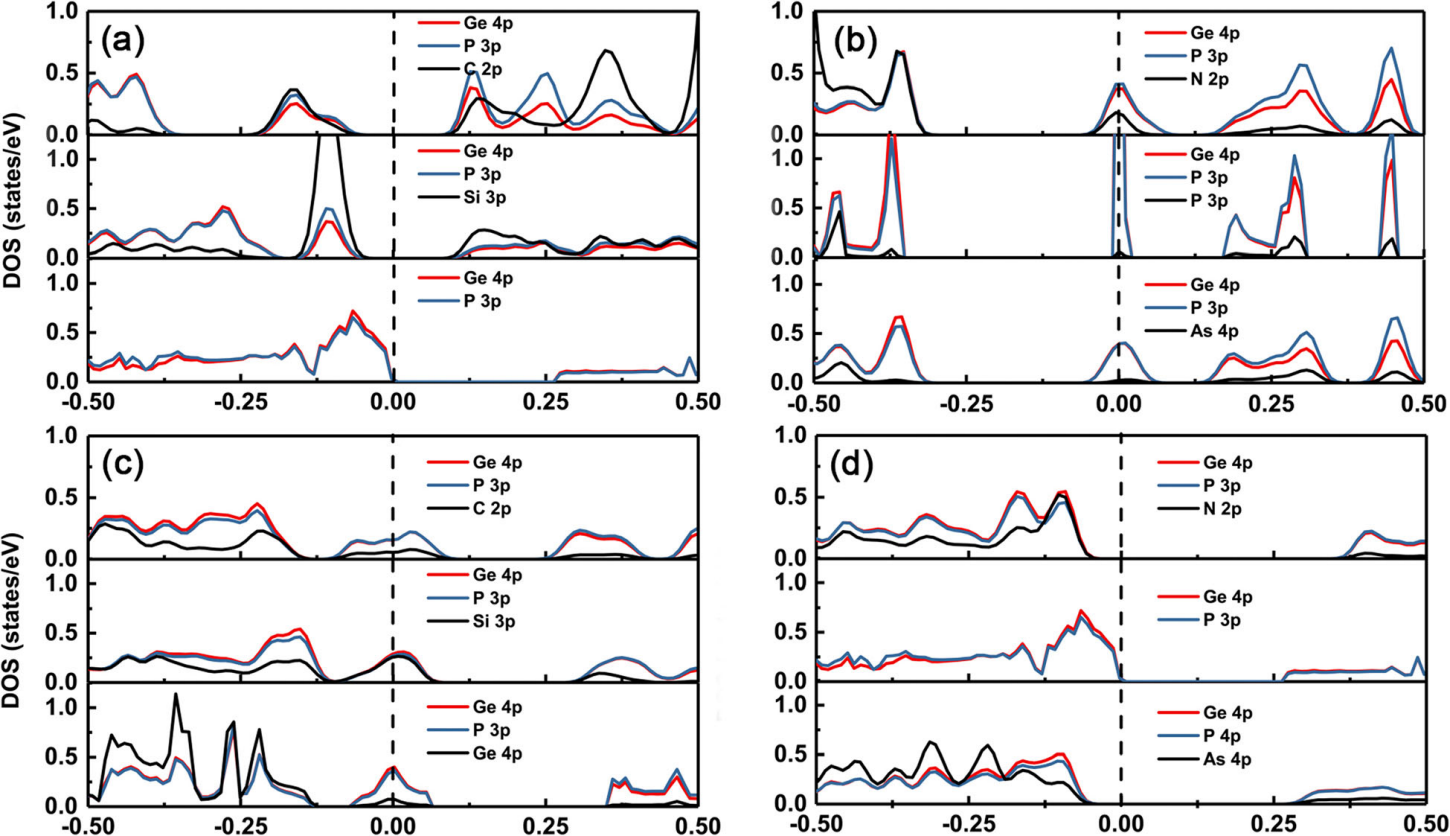
DOS for the doped systems. The partial density of states (right) for group IV (C, Si, and Ge) and group V (N, P and As) atoms doped GeP_3_. The vertical blacked dashed line is the Fermi level. (a) and (b) substituted Ge atom, (c) and (d) substituted P atom

In order to understand the changing of the electronic structures of different dopants in the GeP_3_ monolayer, we plotted the partial density of states (PDOS) of intrinsic and doping group IV–V in GeP_3_ monolayer, as shown in Fig. 5a–d, respectively. It can be seen clearly that the valence band maximal (VBM) and conduction band minimum (CBM) of the GeP_3_ monolayer mainly stems from both p orbitals of Ge and P atoms. When the dopants with the same number of valence electrons as Ge atom, such as C and Si, are available, there will be impurity states just locating above the VBM of intrinsic GeP_3_ monolayer because the p orbital energy level of C and Si are higher than that of the P atom (see Fig. 5a). Therefore, the conductive property is intact and the magnitude of the band gap change is relatively small. However, when the dopants have one more electron than the Ge atom, such as N, P, and As, there will be also impurity states in the band gap and the impurity states originate from the hybridization of splitting CBM (dominant) and the states of dopants (see Fig. 5b).

On the contrary, for the doping on the P site, when the dopants have one less valence electron than the P atom such as group IV, there will be impurity states across the Fermi level, and the impurity states are composed of splitting VBM (dominant) and the states of dopants. Whereas, when the dopants have the same number of valence electrons as P atom such as group V, the doped systems still keep a semiconducting characteristic (see Fig. 5c). The band gaps become relatively larger than the one of intrinsic GeP_3_ monolayer due to the larger mismatch of lattice constants. Moreover, we also observed that the band gap of the N dopant is larger than that of the As dopant in substituting P atoms. This is because the p orbital energy level of the As atom is higher than that of the N atom, thus the higher the energy level p orbital, the more the up shift of impurity states away from VBM (see Fig. 5d).

### Logical Relations for Co-doping Systems

On the basis of the aforementioned findings of single different dopants, we therefore can be able to design co-doping systems to satisfy the electronic properties that we want. Here, we only show the results of B, C, N, and O as examples to illustrate the co-doping effect, but the conclusion is robust against the different selected dopants. For example, on the Ge site co-doping, both the two dopants with one less valence electron can naturally lead to semiconducting property, while for the two dopants with one less and more number of valence electrons, the co-doping systems can thus also have a semiconducting property.

**Fig. 6:**
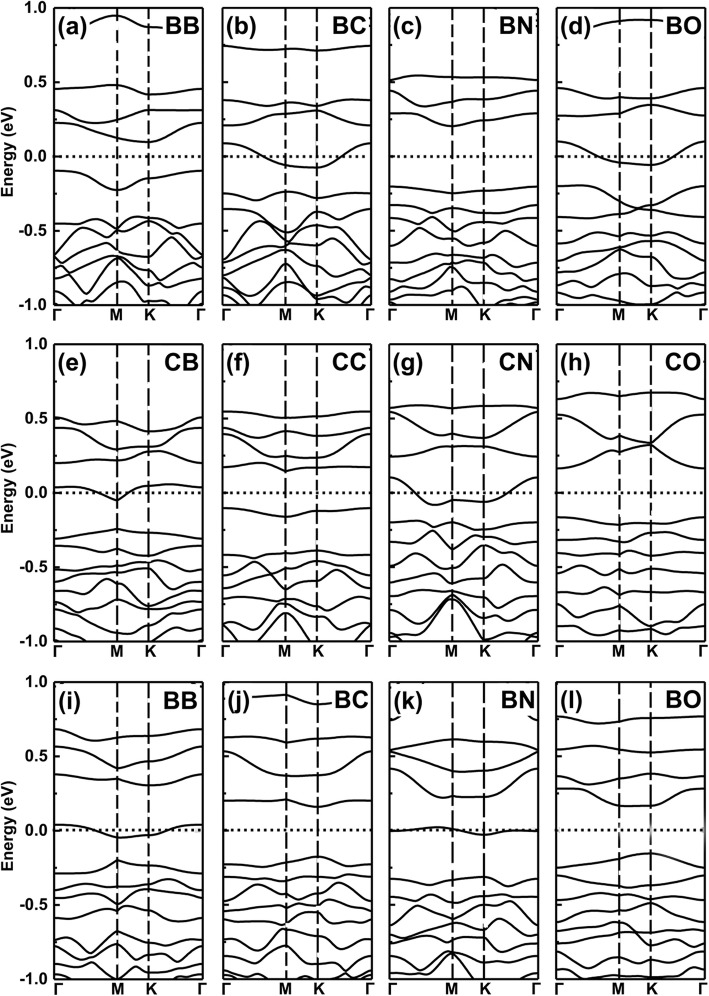
Band structures of co-doped systems. The band structures of B, C, N, and O co-doped GeP_3_ monolayer. **a**–**d** The two dopants substitute two Ge atoms in GeP_3_ monolayer, **e**–**h** the two dopants substitute two P atoms, **i**–**l** the two dopants substitute one Ge atom and one P atom, respectively

However, for the two dopants with one less (more) and the same valence electron, the co-doping systems still keep the metallic property as one less (more) number of valence electron dopants resulted property. Simplify, this idea is exactly confirmed by our further density functional theory (DFT) calculations of co-doped systems, see the results in Fig. 6a–l for the band structures of B, C, N, and O co-doped GeP_3_ monolayer.

**Fig. 7:**
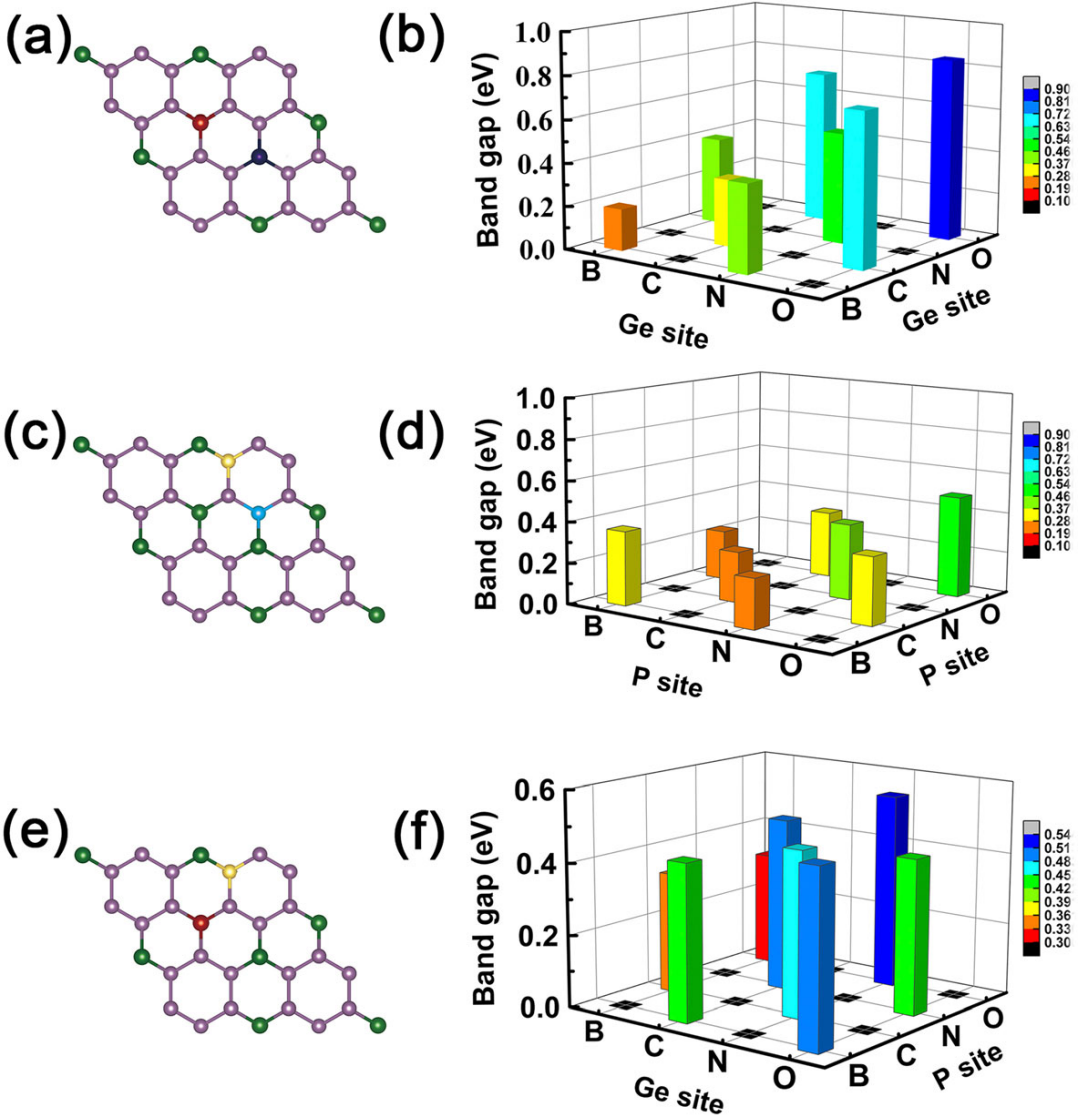
Band gaps of all co-doped systems. The magnitude of band gaps of co-doped GeP_3_ monolayer, the lefts are the sketch of co-doped sites, and the rights are the magnitude of band gaps corresponding to the doping elements. **a**, **b** The case for doping elements occupy the two Ge atoms. **c**, **d** The case for doping elements occupy the two P atoms. **e**, **f** The case for doping elements occupy the Ge and P atoms, respectively

Now, we can give a sample logical-like operation “AND,” setting metallic property as “M” and semiconducting characteristic as “S”. We define the logical relations: M AND M = S, S AND S = S, and M AND S = M, respectively. Herein, these findings we have obtained above obey such logical-like relations, for instance, the dopants with one more and one less valence electron result to metallic property, but when we use the two dopants as co-doping, such as B and N on Ge sites as shown in Fig. 7a and b, the co-doped systems become semiconducting property as expected see Fig. 6c. If we choose B-C co-doped GeP_3_ monolayer system, it presents metallic feature which is the case of M AND S (see Fig. 4a, b). The same for C-N, N-O, and B-O atoms co-doping in GeP_3,_ substituting two Ge atoms, two P atoms, or one Ge and P atoms, as shown in Fig. 7c–f, respectively.

**Fig. 8:**
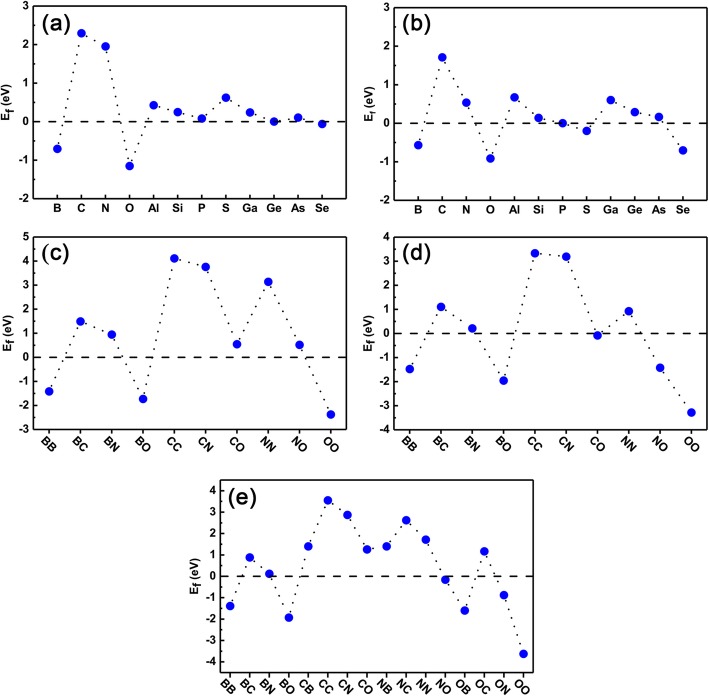
Formation energy of all doped systems. The calculated formation energy of single element doping and co-doping systems. **a**, **b** are the dopant-substituted Ge atom and P atom, respectively; **c**–**e** The co-dopants substituted by two Ge atoms, two P atoms, and one Ge atom and one P atom, respectively

Finally, we checked the stability of both the single-doped systems and co-doped systems for ensuring whether they can be realized further in the experiment. The formation energy is calculated using the Eqs. (1) and (2) for single-doping and co-doping cases, respectively. The results are present in Fig. 8a–e. From Fig. 8a and b, we can see clearly that the *E*_*f*_ of the single dopants in Ge sites are all close to that of GeP_3_ monolayer (setting to zero as a reference point), excepting C, N, and S atoms. We also noticed that, for dopants of B, O, P, Ge, and Se atoms, the formation energies are much smaller than other dopants, indicating they are very easy for doping in an experiment. For dopants in the P sites, dopants of B, O, P, and Ge atoms have relatively smaller formation energy and they are also easy for doping. C, N, Al, and Ga are not easy for doping.

As for the formation energy of co-doping, Fig. 8c–e are the formation energy of co-doping with where two dopants occupy the positions of two Ge atoms (denoted as Ge-Ge sites), two P atoms (denoted as P-P sites), one Ge and one P atoms (denoted as Ge-P sites), respectively. Here we only show the results of dopants of B, C, N, and O as an example. For Ge-Ge sites and P-P sites the formation energy of co-doping can be estimated roughly by averaging the formation energies of single-element doping separately. Clearly, for BB, BO, and OO co-doping in Ge-Ge sites and BB, BO, NO, and OO co-doping in P-P sites, the formation energies are relatively small and can be realized easily in the experiment. However, for CC, CN, and NN co-doping in Ge-Ge sites and CC and CN co-doping in P-P sites, the formation energies are relatively larger, indicating they are difficult to dope in experiment. For Ge-P sites co-doping, as shown in Fig. 8e, the formation energy becomes more complex than Ge-Ge or P-P sites co-doping because there is charge transfer between the dopants. In any wise, the BB, BO, and OO co-doping have the smaller formation energies, while CC, CN, and NN co-doping have larger formation energies. In general, the formation energy highly depends on the number of valence electrons of dopant. Specifically, when the two dopants with one less (more) electron than the substituted atoms, the formation energy of co-doped system is lower (higher) than that of the corresponding single dopants such as BB (NN) co-doped Ge sites. This is because there exists competition between the decreased (increased) energy of reduced (increased) electrons of doped systems and Coulomb repulsion. For the hole-hole co-doping, the energy of the former case is much larger than the latter case thus resulting to the quite decreased formation energy in co-doping systems such as BB, while for the electron-electron co-doping, both the former and later cases lead to the higher formation energy such as NN. However, for hole and electron co-doped systems such as BN co-doped Ge sites the formation energy is dramatically lower than the corresponding single-doped cases. This is because in such co-doped system, there is no energy gain from net added or reduced electrons in the systems, and the Coulomb interaction plays a decisive role in the forming co-doped dopants. In all, taking together our previous studies of element doping in black phosphorene, it is should be pointed out that our present studies have a certain degree of universality and are expected for applying other 2D semiconductor monolayers, such as BN, MoS_2,_ and so on.

**Fig. 9:**
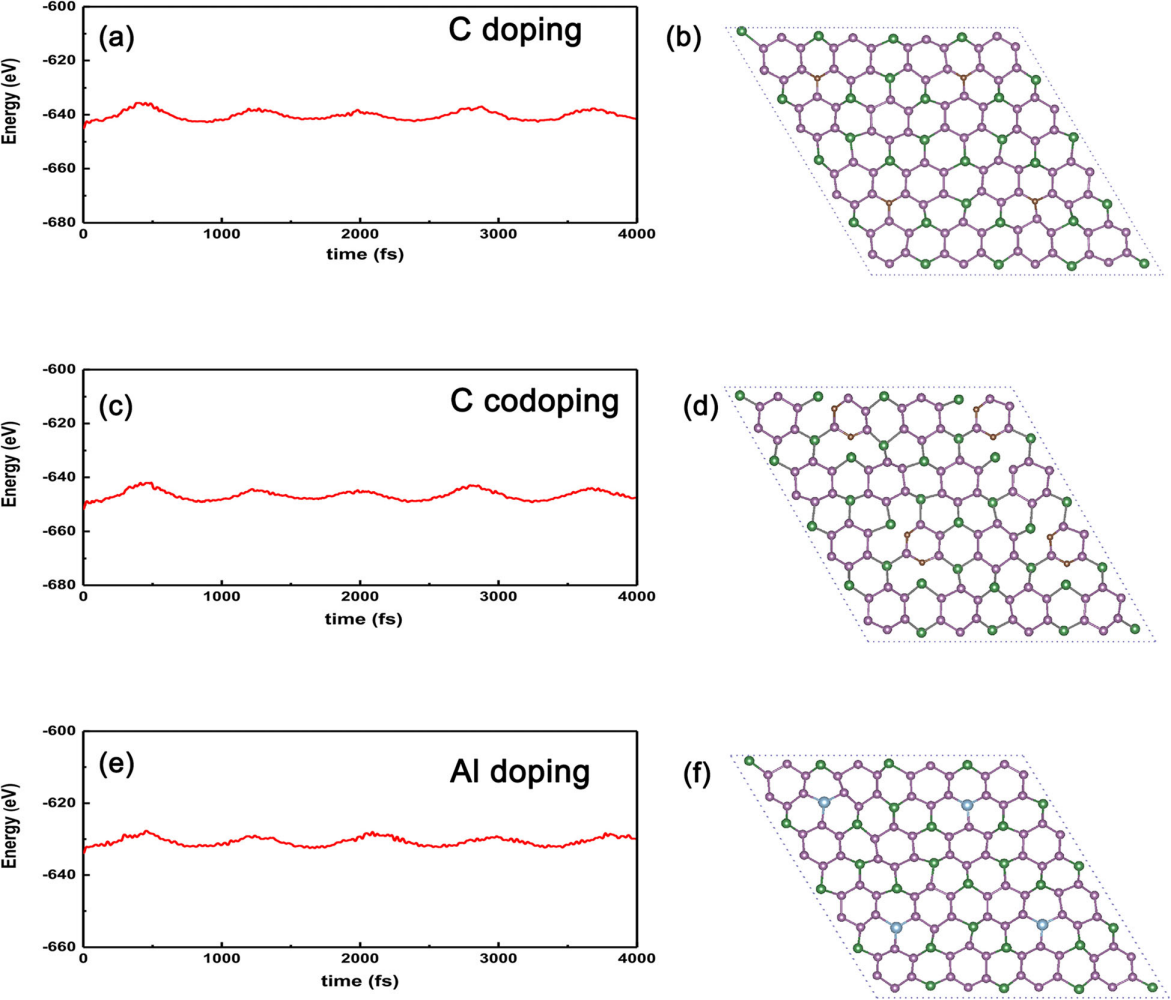
AIMD for the C, two C, and Al atom-doped systems. AIMD confirms the thermal stability of **a** C atom-doped GeP_3_ with substituting Ge atom, **c** two C atoms doped GeP_3_ with substituting two P atoms, and **e** Al atom-doped GeP_3_ with substituting at 300 K. The structures in **b** C, **d** two C atom, and **f** Al correspond to their final structures after 4000 fs

Finally, to check the stability of the above doped systems compared with the un-doped case, we carried out the AIMD (ab initio molecular dynamics) to show the energy vs time, as shown in Fig. 9a–f. We can see clearly that the oscillatory amplitude will be convergent as long as the time is lasting enough (~ 4 ps), implying that the doped systems will not collapse against thermal fluctuation up to 300 K for C-doped GeP_3_ in Fig. 9a. Even for the most active C atom-doped GeP_3_, the extreme temperature can be up to 300 K, as shown in Fig. 9c. Besides, we also take metal Al substitution on Ge site as an example, the calculated result is shown in Fig. 9e and f, from which we can see clearly that the energy oscillating amplitude gradually decreases with the time lasting, meaning that the energy could be convergent as long as the time lasting enough and the structure of the doped system is thermally stable against thermal fluctuation. Therefore, we can expect that such doped systems can be realized in further experiments given the high-quality GeP_3_ monolayer is prepared.

In the end, we want to discuss the reliability of our study presented here. Our conclusions presented here are predicted results theoretically but are highly reliable. This is because our host material used here has been reported and its bulk phase of layered GeP_3_ already exists [26]. So our studied doping-induced related phenomena need to be further confirmed in the experiment once monolayer GeP_3_ is realized further. Then, doping the corresponding atoms could be conducted. For simplicity, doping electron or hole in monolayer GeP_3_ could be realized by the adsorption of some molecules.

## Conclusion

In summary, we have investigated the electronic properties of group III to VI dopants in 2D GeP_3_ monolayer and find that the doped GeP_3_ with substitution on Ge site exhibits metal-semiconductor oscillations as a function of the number of valence electrons of dopants, while such oscillations are reversed with substitution on P site. Based on the results of single dopants, we could propose the conducting properties of co-doping in GeP_3_, which can be obtained by a simple logical operation. Finally, we calculate the formation energies of various dopants and find that some of the co-doped systems, especially for the electron-hole and hole-hole co-doping, are more energetically favorable because of the Coulomb attraction. Our findings not only present a new phenomenon but also propose an intriguing route to tune the electronic properties in 2D binary semiconductors.

## Data Availability

The datasets generated during and/or analyzed during the current study are available from the corresponding author on request.

## Abbreviations

1D: One dimensional
2D: Two dimensional
AIMD: Ab initio molecular dynamics
BP: Black phosphorene
CBM: Conduction band minimum
DFT: Density functional theory
HSE06: Hybrid density functional
PBE: Perdew-Burke-Ernzerhof
PDOS: Partial density of states
VBM: Valence band maximum
